# The Influence of Critical Shoulder Angle in Secondary Rotator Cuff Failure After Shoulder Replacement: An Age- and Sex-Matched Case-Control Study

**DOI:** 10.7759/cureus.19277

**Published:** 2021-11-05

**Authors:** Joshua L Filer, Damien Tucker, Partha Sarangi, Phil McCann

**Affiliations:** 1 Trauma and Orthopaedics, University Hospitals Bristol and Weston NHS Trust, Bristol, GBR; 2 Trauma and Orthopaedics, North Bristol NHS Trust, Bristol, GBR

**Keywords:** shoulder replacement, shoulder, revision surgery, shoulder arthroplasty, secondary rotator cuff failure, csa, critical shoulder angle

## Abstract

Introduction

Decreased or increased critical shoulder angles (CSA) are associated with osteoarthritis or rotator cuff failure respectively. Secondary cuff failure after shoulder arthroplasty is disabling and often requires additional surgery. The aim of this study was to investigate if the initial CSA correlated with cuff failure in the context of shoulder arthroplasty.

Methods

Patients from a tertiary referral centre were reviewed from 2011-2017. Those who underwent revision from hemiarthroplasty (HA) or anatomic total shoulder arthroplasty (TSA) to a reverse shoulder arthroplasty (RSA) following rotator cuff failure were compared to an age and sex-matched control group. The CSA was calculated from initial pre-operative radiographs.

Results

In this study, 16 patients with symptomatic cuff failure after anatomic TSA or HA requiring revision to RSA were compared to a control group of 16 age- and sex-matched patients showing no signs of symptomatic cuff failure. The median CSA in the study group was significantly greater than that of the control group (31.5^o^, IQR = 29.8 - 36.1^o^ vs. 29.5^o^, IQR = 27.6 - 30.4^o^; p= 0.026).

Conclusion

The difference in CSA between those who required revision for secondary cuff failure than those who didn’t suggest that pre-operative measurement of CSA may help guide surgical planning in shoulder arthroplasty.

## Introduction

Shoulder arthroplasty including anatomic total shoulder arthroplasty (TSA) and hemiarthroplasty (HA) are established treatments for glenohumeral osteoarthritis. Secondary rotator cuff failure following shoulder arthroplasty is a recognised complication that can lead to the need for revision surgery [1-3]. The rates of revision as a result of secondary cuff failure are variable with studies reporting rates between 1% and 17% [1, 4-6]. Data from the UK National Joint Registry (NJR) showed that 26% of revision shoulder replacements were performed due to rotator cuff failure in 2018 [7]. Rotator cuff tears following shoulder arthroplasty can result in pain, instability, weakness and decreased range of motion, leading to impairment of function [8]. Given the associated morbidity and financial cost of revision shoulder arthroplasty, it is important to reduce the need for revision as far as possible [9, 10].

Identifying those who may be at risk of cuff failure pre-operatively may help to reduce the risk of revision arthroplasty. Various anatomical characteristics of the shoulder such as acromial morphology and glenoid orientation (inclination and version) have been investigated and found to be associated with rotator cuff pathology [11-17]. Expanding on these concepts, Moor et al. introduced a radiographic measure of shoulder morphology that integrated both glenoid inclination and lateral extension of the acromion [18], which were known risk factors for rotator cuff pathology [14, 16-20]. They termed this measure the Critical Shoulder Angle (CSA) and found that it could be correlated with shoulder girdle pathology including rotator cuff tears and glenohumeral osteoarthritis [18]. This study found that a larger CSA (>35-38^o^) was associated with rotator cuff tears, whereas a smaller CSA (<28^o^) was associated with osteoarthritis [18]. Subsequent research validating this finding suggests that shoulders with a smaller CSA are exposed to greater compressive glenohumeral joint forces, predisposing to osteoarthritis [21]; whereas those with a larger CSA require greater supraspinatus activity to maintain stability leading to an overload of the muscle-tendon unit predisposing to rotator cuff tears [22, 23].

Previously, others have tried to establish whether CSA is associated with the risk of revision due to rotator cuff failure [3]. However, despite showing a non-significant trend for higher CSA values in those undergoing revision compared to controls (33^o^ vs 32^o^, p = 0.956) [3], the approach to measuring CSA in these patients may have been flawed, as the measurement of CSA was performed on the radiographs of patients with implants in situ rather than in the pre-operative native joint radiographs.

The aim of this study was to further evaluate the usefulness of measuring the pre-operative CSA of patients undergoing shoulder arthroplasty in predicting potential secondary rotator cuff failure and the need for revision surgery. We hypothesised that patients who have undergone shoulder arthroplasty and subsequent revision due to secondary rotator cuff failure would have greater pre-operative CSA values, than those who did not require revision arthroplasty.

## Materials and methods

Inclusion criteria

In this case-control study, the medical records and imaging studies of all patients who underwent shoulder HA or anatomic TSA at our institution between 2011 to 2017 were retrospectively reviewed. All those who underwent revision to reverse shoulder arthroplasty (RSA) were identified. The reasons for revision were investigated and determined where possible from the medical records and NJR data. Patients were included in the study group if they underwent a revision for rotator cuff failure/deficiency. The radiographs of these patients were reviewed and those with inadequate radiographs were excluded. These included patients for whom there were no pre-operative radiographs available or if the radiographs had more than 20 degrees of rotation, as determining the CSA on such images is known to be unreliable [18, 24]. Of these patients, over 80% (13/16) underwent their primary arthroplasty for glenohumeral osteoarthritis. An age- and sex-matched group of patients who had also undergone shoulder arthroplasty (TSA or HA) for osteoarthritis but had not yet undergone any revision surgery were identified as the control group. We matched controls to cases in a 1:1 ratio. All patients provided consent for their data to be used and Institutional Review Board approval to conduct this study was granted by the University Hospitals Bristol NHS Foundation Trust, which does not require ethical approval for reporting individual cases or case series.

Measurements

The CSA for both the study and control groups was calculated independently by two of the authors (JF and DT) using the available pre-operative anteroposterior radiographs. This was achieved using the in-built measurement tools of the picture archiving and communication system (PACS) software (InSight PACS, Insignia Medical Systems and SYNAPSE PACS, Fujifilm).

**Figure 1:**
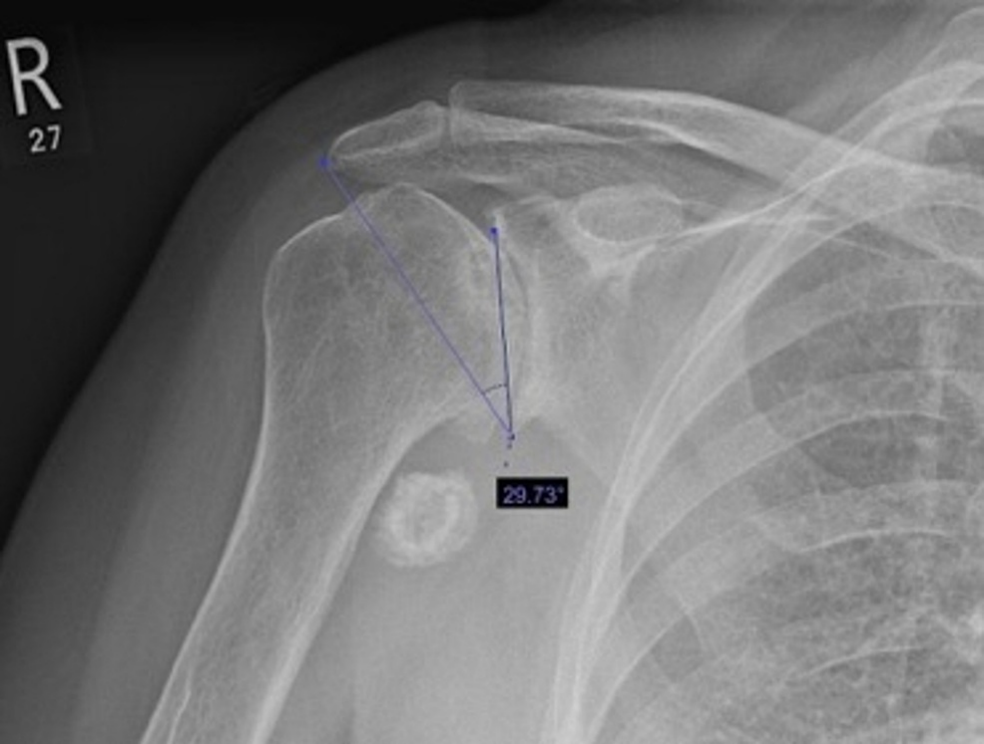
Measurement of the critical shoulder angle (CSA) on an anterior-posterior radiograph of the right shoulder. (CSA is measured as the angle between a line parallel to the glenoid, and a line through the inferior-lateral edge of the glenoid and the inferior-lateral edge of the acromion, in this example, the angle was 30^o^)

As described by Moor et al. [18], the angle was formed by a line connecting the superior and inferior bony margins of the glenoid and a line drawn from the inferior bony margin of the glenoid to the infero-lateral edge of the acromion (Figure 1).

To assess intra- and inter-reliability of the measurement of CSA all radiographs for all subjects were independently reviewed by two of the authors (JF and DT) and measurements were taken on two occasions each, with repeated measurements taken at least a week apart and the findings of each author blinded to the other, and themselves on each occasion. 

Statistical analyses

All statistical analyses were performed using SPSS® Statistics v. 27 (IBM Corp., Armonk, NY). As the CSA was a continuous variable we checked the two groups for normality of distribution using the one-sample Kolmogorov-Smirnov test. An independent samples Mann-Whitney U test was used to compare the pre-operative CSA for cases and controls. For intra- and inter-observer reliability of the measurement of the CSA in both cases and controls, intra-class correlation coefficients (ICC, two-way mixed effect models) were calculated. An ICC-score of < 0.40 was considered poor, 0.40-0.59 = fair/moderate, 0.60-0.74 = good and ≥ 0.75 = excellent [25]. This was supplemented by visual inspection of Bland-Altman plots, showing the difference between the two measurements against their mean. Reliability is indicated visually with at least 95% of all dots being within the upper and lower limits of agreement [26, 27].

## Results

**Figure 2:**
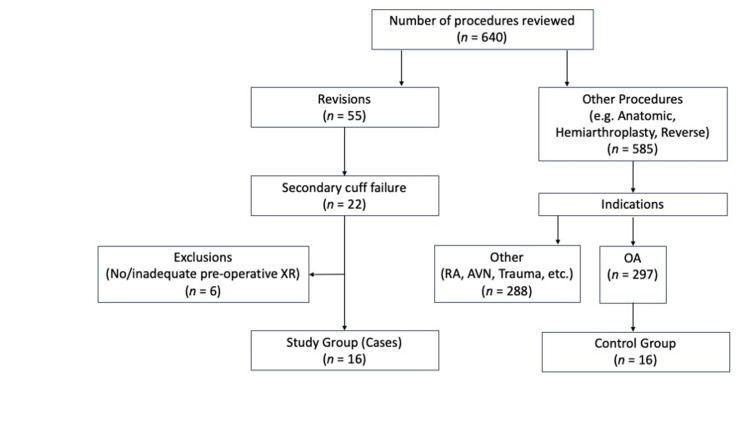
Study flow chart in line with the STROBE (Strengthening the Reporting of Observational Studies in Epidemiology) statement. Study group: revision cases due to secondary rotator cuff failure; control group: an age- and sex-matched group of cases of primary shoulder arthroplasty. RA: rheumatoid arthritis, AVN: avascular necrosis; XR: X-ray; OA: osteoarthritis.

In our institution, a total of 640 shoulder arthroplasties were performed on 566 patients between 2011 and 2017. Of these, 55 were revision procedures for a variety of reasons including instability, component loosening or wear, infection and rotator cuff failure. Within the revision procedures, 22 were a revision to RSA as a result of secondary rotator cuff failure and thus eligible for inclusion in the study group (Cases). Of these cases, six were excluded as there were inadequate or absent pre-operative radiographs (Figure 2).

Of the 16 cases in the study group, nine originally were anatomic TSA and seven were previously HA. Prostheses used included the Arthrex Eclipse (Arthex, Naples, FL), Synthes Epoca (DePuy Synthes, Raynham, MA) and Zimmer-Biomet Copeland systems (Zimmer-Biomet, Warsaw, IN).

The study group consisted of 16 cases, but there were 14 individual patients as two underwent bilateral revision shoulder arthroplasty. Nine of these patients were female and five were male. Of the bilateral revisions, one was male and another was female. The median age at the time of surgery was 75.5 years (range 60-86). The average time to revision was four years (range six months - nine years). For the control group, there is a minimum of three years' follow-up, with an average of six years. In terms of demographics the control group and the study group were comparable (Table 1).

**Table 1:** Study group demographics

	Controls	Revisions
Number of patients	16	14
Number of procedures	16	16
Age		
Median	73.5	75.5
Range	60-85	60-86
Gender		
Male	6 (38%)	6 (38%)
Female	10 (62%)	10 (62%)
Laterality		
Left	10 (62%)	8 (50%)
Right	6 (38%)	8 (50%)
ASA		
Mode	2	3
Range	1-3	1-4

**Figure 3:**
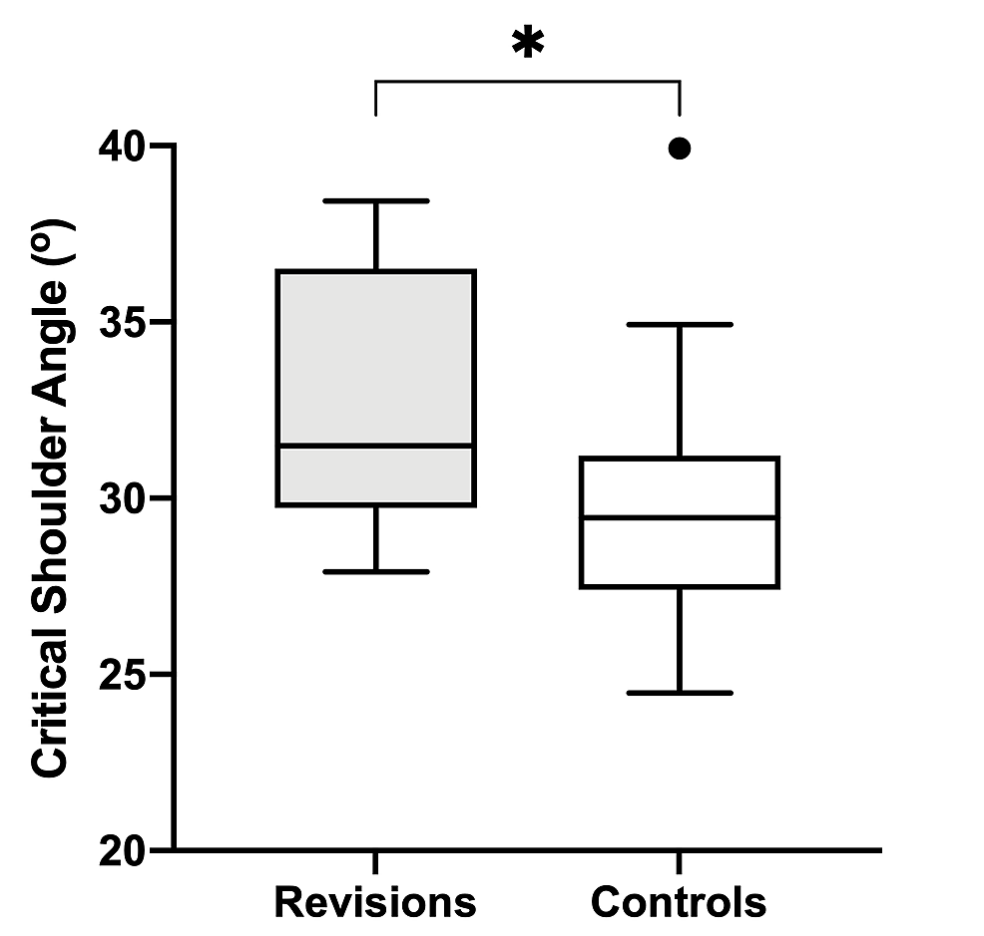
Comparison of the median critical shoulder angle (CSA) in controls and revisions due to secondary rotator cuff failure. Boxplots show the median and interquartile range (IQR); whiskers represent the range of critical shoulder angle (CSA), outliers are shown as circles. The asterisk represents a statistically significant difference (*p* = 0.026).

The median pre-operative CSA in the study group was 31.5^o^ (interquartile range, IQR = 29.8-36.1^o^), compared to the control group where the median was 29.5^o^ (IQR = 27.6 - 30.4^o^). The study group CSA was found to be significantly greater than the control group (U = 187, difference = 2^o^, 95% CI of difference = 0.25 - 5.38, *p* = 0.026; Figure 3).

**Figure 4:**
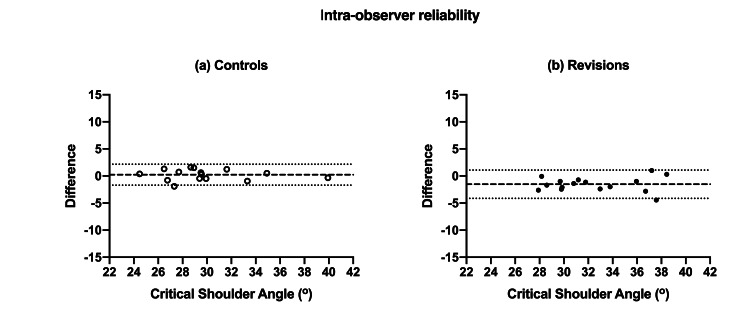
Bland-Altman plots for intra-observer reliability of CSA measurement of controls (A) and revisions (B).

**Figure 5:**
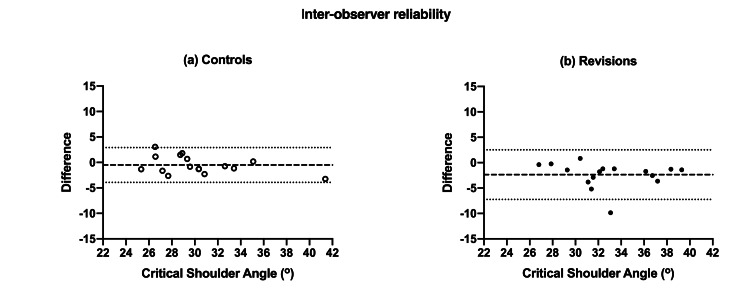
Bland-Altman plots for inter-observer reliability of CSA measurements of controls (A) and revisions (B).

Intra-observer reliability of CSA measurement showed excellent agreement for both controls and cases with ICC of 0.982 (95% CI; 0.951-0.994) and 0.927 (0.331-0.982) respectively (both statistically significant with p <0.0001). Interobserver reliability was also excellent with ICC of 0.951 (0.863-0.983) for controls and 0.809 (0.137-0.944) for cases (both p <0.0001). This inter-observer agreement was also statistically significant (p <0.0001). The intra- and inter-observer reliability of measurements is also confirmed visually using Bland-Altman plots (Figures 4-5 respectively).

## Discussion

The results of our study demonstrate that those patients undergoing revision shoulder arthroplasty for secondary rotator cuff deficiency following HA or anatomic TSA have significantly greater pre-operative CSA values than similarly aged and sex-matched controls who have not undergone revision shoulder arthroplasty after their initial HA or anatomic TSA.

Our findings are consistent with those of previous studies that have investigated CSA in individuals who have rotator cuff pathology with CSA measures that are larger than in those who have osteoarthritis [18, 28]. The controls in our study all had osteoarthritis without rotator cuff dysfunction and, as expected, the average CSA was similar to the values found by Moor et al. for patients with osteoarthritis [18]. Although our revision group had average CSA values greater than controls, our value of 33^o^ is expectedly lower than the 38^o^ previously found for people with rotator cuff pathology in the aforementioned study and indeed is similar to their control healthy population [18]. Although CSA values in the region of 32-35^o^ may commonly be found in normal healthy shoulders based on Moor’s study [18], other studies have also found values similar to ours in abnormal shoulders. For example, Mantell et al. found that those with concurrent rotator cuff tear and osteoarthritis had CSA values of approximately 35^o^ [18, 28]. These results highlight that there may be a grey area of CSA values that could be considered normal, but could also be abnormal. Our results suggest that patients with values in this range may be at risk of secondary rotator cuff failure following TSA or HA. In these patients considering primary RSA over TSA or HA may lead to fewer revisions.

Although our findings show that there is a significantly greater pre-operative CSA among people who subsequently go on to require revision for secondary rotator cuff failure than those who do not, other similar studies have not found this. In particular, Cerciello et al. reported a study of patients who had experienced secondary rotator cuff failure following shoulder arthroplasty [3]. In this study, they showed that there may have been a trend for greater CSA values amongst those undergoing revision than age and sex-matched controls who did not experience secondary cuff failure, but that this was not significant [3]. Just as in our study there were small numbers of cases, which may have hindered their ability to detect a significant difference between groups. Although their conclusions are contradictory to ours, the methods for measuring CSA were different between the studies. In their study, CSA was measured on shoulders with prostheses in situ just prior to their revision and after their index arthroplasty. Our study evaluated the CSA for native shoulders just prior to the index arthroplasty. It may be that the anatomical morphology of their subjects was altered by their index surgery either intentionally or otherwise. Any correction of the morphology of the shoulder joint that may have occurred, such as a change of glenoid inclination (and hence CSA), may not be sufficient to mitigate the risks of having abnormal anatomy for prolonged periods prior to index arthroplasty. This prolonged exposure to abnormal bony anatomy may have caused irreversible damage to the rotator cuff and led to failure even if the CSA was improved following primary arthroplasty.

A finding that is shared with the above study by Cerciello et al. [3], was the reliability of the measurement of the CSA. They found ICC values of 0.956 showing excellent inter-observer agreement in measurements of CSA [3]. This reliability has been clearly documented in many previous studies measuring CSA and shows the utility of using such measures of anatomical differences when radiographically assessing shoulder joints for potential pathology [18, 28-30].

Study limitations

This study had some limitations. As with other studies of revision shoulder arthroplasty following rotator cuff failure [3], there was a low revision rate at our institution, leaving only a small number of cases to assess. The small number of cases including measures on both shoulders in two patients from the study group limits the power of the study. 

In addition to reducing the statistical power of a study, a small sample size also raises the possibility of unintentional selection bias. A further risk for unintentional selection bias arises from the fact that we do not know if there were patients who had rotator cuff dysfunction but did not go on to revision as a result of other factors such as co-morbidities or patient choice.

Furthermore, not all patients diagnosed with secondary cuff failure had this clinical diagnosis confirmed with either pre-revision imaging (MRI) or documented intra-operative confirmation of the details of rotator cuff failure. This leads to the possibility that rotator cuff failure may have been overestimated. Conversely, it is also possible that some patients who were revised for pain, also had undocumented rotator cuff failure, but as this was not identified they were not included in the study.

Despite these limitations, the findings of this study suggest that CSA is an important factor involved in secondary rotator cuff failure following shoulder arthroplasty, and should be taken into consideration when planning such procedures.

## Conclusions

In our series, the pre-operative CSA was greater in patients who had undergone shoulder replacement and experienced secondary cuff failure necessitating revision arthroplasty compared to those who did not develop cuff failure warranting revision surgery. Although our sample size was small, CSA measurement may be useful during planning shoulder arthroplasty and help to guide the choice of technique to be performed in order to reduce the risk of later revision. To verify the findings of this study, further prospective, multi-centre studies are needed. As the number of patients undergoing shoulder arthroplasty continues to increase, the revision burden will also rise. Therefore, a greater understanding of the factors that affect implant survivorship is paramount. A clearer insight into the effect of the CSA in shoulder arthroplasty may better equip the surgeon in deciding which prosthesis best fits the individual pathology.
